# The role of Neutrophil counts, infections and Smoking in mediating the Effect of Bronchiectasis on Chronic Obstructive Pulmonary Disease: a mendelian randomization study

**DOI:** 10.1186/s12890-024-02962-6

**Published:** 2024-03-20

**Authors:** Lei Gu, Wei Liu, Jian-an Huang, Lujian Zhu, Xiaowen Hu, Jian Yue, Jing Lin

**Affiliations:** 1 Department of Pulmonary and Critical Care Medicine, The First Affiliated Hospital of Soochow University, Suzhou, 215006 China; 2 Institute of Respiratory Diseases, Soochow University, Suzhou, 215006 China; 3 Respiratory Diseases, Suzhou Key Laboratory, Suzhou, 215006 China; 4 Department of Respiratory and Critical Care Medicine, The 900th Hospital of Joint Logistic Support Force, People’s Liberation Army, Fujian Medical University, Fuzhou, 350025 China; 5 Department of Infectious Diseases, Affiliated Jinhua Hospital, Zhejiang University School of Medicine, Jinhua, 321000 China; 6 Department of Pulmonary and Critical Care Medicine, The First Affiliated Hospital of USTC, Division of Life Sciences and Medicine, University of Science and Technology of China, Hefei, Anhui, 230001 China; 7 The People’s Hospital of Gaozhou, Gaozhou, 525200 China; 8 Department of Infectious Diseases, The First Affiliated Hospital of Soochow University, Suzhou,Jiangsu Province, 215006 China

**Keywords:** Bronchiectasis, Chronic obstructive Pulmonary Disease, Neutrophil, Respiratory infections, Smoking, Mendelian randomization

## Abstract

**Background:**

The causality of the relationship between bronchiectasis and chronic obstructive pulmonary disease (COPD) remains unclear. This study aims to investigate the potential causal relationship between them, with a specific focus on the role of airway inflammation, infections, smoking as the mediators in the development of COPD.

**Methods:**

We conducted a two-sample Mendelian randomization (MR) analysis to assess: (1) the causal impact of bronchiectasis on COPD, sex, smoking status, infections, eosinophil and neutrophil counts, as well as the causal impact of COPD on bronchiectasis; (2) the causal effect of smoking status, infections and neutrophil counts on COPD; and (3) the extent to which the smoking status, infections and neutrophil counts might mediate any influence of bronchiectasis on the development of COPD.

**Results:**

COPD was associated with a higher risk of bronchiectasis (OR 1.28 [95% CI 1.05, 1.56]). Bronchiectasis was associated with a higher risk of COPD (OR 1.08 [95% CI 1.04, 1.13]), higher levels of neutrophil (OR 1.01 [95% CI 1.00, 1.01]), higher risk of respiratory infections (OR 1.04 [95% CI 1.02, 1.06]) and lower risk of smoking. The causal associations of higher neutrophil cells, respiratory infections and smoking with higher COPD risk remained after performing sensitivity analyses that considered different models of horizontal pleiotropy, with OR 1.17, 1.69 and 95.13, respectively. The bronchiectasis–COPD effect was 0.99, 0.85 and 122.79 with genetic adjustment for neutrophils, respiratory infections and smoking.

**Conclusion:**

COPD and bronchiectasis are mutually causal. And increased neutrophil cell count and respiratory infections appears to mediate much of the effect of bronchiectasis on COPD.

**Supplementary Information:**

The online version contains supplementary material available at 10.1186/s12890-024-02962-6.

## Introduction

Chronic obstructive pulmonary disease (COPD) stands out as the most widespread and clinically significant chronic respiratory disease worldwide. It is characterized by airflow limitation, persistent airway inflammation, and a gradual decline in lung function [1]. Globally, the estimated prevalence of COPD is approximately 13.1% [2], and it ranks as the third leading cause of death, claiming the lives of approximately 3.2 million individuals annually [3]. The presence of bronchiectasis, as determined through radiological evidence on computed tomography scans in individuals with COPD, displays a substantial range in occurrence, ranging from 4–72% [4]. Both COPD and bronchiectasis share the common trait of chronic airway inflammation, which leads to significant morbidity and mortality. Although COPD and bronchiectasis exhibit distinct clinical and pathological features, there is a growing acknowledgment of their interconnection, particularly regarding shared risk factors and overlapping inflammatory pathways.

Bronchiectasis has been identified as a potential predisposing factor for the development of COPD, although the precise nature of their relationship and the underlying mechanistic pathways remain incompletely elucidated. Observational studies have consistently revealed a heightened likelihood of COPD development in individuals with a history of bronchiectasis, hinting at a potential causal connection [5, 6]. Nevertheless, further exploration is required to unveil the mechanisms by which bronchiectasis might contribute to the initiation and progression of COPD.

Bronchiectasis and COPD are increasingly recognized as part of a spectrum of diverse obstructive airway diseases, each with distinct characteristics [4, 7]. Considerable evidence points to the involvement of various immune mediators in the development of bronchiectasis and COPD, with a significant emphasis on blood cells. Recent findings indicate that specific components of the immune response, such as neutrophils, play crucial roles in the pathogenesis of both bronchiectasis and COPD [8]. Eosinophils, known for their role in allergic inflammation, are not only strongly associated with the severity of asthma and COPD but also exhibit a U-shaped relationship with the severity and exacerbations in patients with bronchiectasis [9]. Neutrophils, central to innate immunity, are implicated in the airway inflammation observed in COPD, asthma, and bronchiectasis. Notably, in COPD, essential inflammatory cytokines and proteases, including neutrophil elastases, have been identified at elevated levels in cases of bronchiectasis [10]. While both bronchiectasis and COPD share the common feature of chronic respiratory tract inflammation, the precise role of these immune cells in mediating the relationship between the two conditions remains uncertain. Of course, age, gender, smoking status and respiratory infections are still common risk factors in bronchiectasis and COPD.

Unlike observational associations, genetic associations are not influenced by classical confounding or reverse causation, as genes are randomly inherited at conception. Mendelian randomization (MR), a potent method that employs genetic variants as instrumental variables (IVs), provides a unique opportunity to investigate causal relationships between exposures and outcomes, which overcomes the traditional effects of numerous confounding factors and reverse causality bias in epidemiological studies, exploring the causal relationships involved [11]. Therefore, the MR approach can provide indirect evidence for a causal link between bronchiectasis and COPD risk, given that the foundational assumptions are satisfied [11]. In this study, our objective is to leverage MR to explore the potential causal connection between bronchiectasis and COPD, shedding light on the role of bronchiectasis as a potential risk factor for the development of COPD.

Therefore, our study also aims to investigate whether eosinophil and neutrophil cell counts, age, gender, smoking status and respiratory infections serve as intermediaries in the observed link between bronchiectasis and COPD. By utilizing genetic variants as instrumental variables for these cellular traits, our objective is to gain insights into the potential biological mechanisms that contribute to the association between bronchiectasis and COPD. If a direct causal relationship indeed exists between bronchiectasis and COPD, it would be reasonable to anticipate that genetic variants increasing the risk of bronchiectasis would likewise elevate the risk of COPD. Insights derived from genetics can, therefore, offer a supplementary perspective for better understanding the biological underpinnings of both conditions [12].

In summary, this study aims to address the current gap in our understanding of the causal relationship between bronchiectasis and COPD by employing MR to establish robust evidence. Additionally, we explore the potential mediating roles of eosinophil and neutrophil cell counts, age, gender, smoking status and respiratory infections in the association between bronchiectasis and COPD, providing a comprehensive grasp of the underlying mechanisms that connect these two clinically significant respiratory conditions.

## Methods

We employed a two-sample MR approach utilizing publicly available datasets that provide genome-wide association results for bronchiectasis, blood cell traits, biological sex (age adjusted), smoking status, COPD/asthma/ILD related infections and COPD. Two-sample MR involves the use of distinct datasets or samples to establish the gene–risk factor associations (e.g., bronchiectasis) and the gene–outcome associations (e.g., COPD). Our analysis followed a two-step MR procedure. In the first step, we examined the causal impact of bronchiectasis on potential mediators, and in the second step, we assessed the causal influence of these potential mediators on COPD.

### Data sources

We sourced relevant data about bronchiectasis, COPD, biological sex (age adjusted), smoking status, COPD/asthma/ILD related infections and blood cells from the IEU Open GWAS project (https://gwas.mrcieu.ac.uk/) and then analyzed GWAS summary-level data. Detailed information about the data sources and sample sizes used in this study is provided in Supplementary Table 1.

### SNP selection criteria

We selected single-nucleotide polymorphisms (SNPs) associated with bronchiectasis, COPD, and blood cell traits as IVs. To explore potential causal relationships between bronchiectasis, blood cell traits, and COPD, we established a genome-wide significance threshold of *p* < 5 × 10^− 8^ or *p* < 5 × 10^− 6^ for identifying IVs. To mitigate potential bias arising from strong linkage disequilibrium (LD), we implemented a clumping algorithm with a cutoff of r^2^ = 0.001 and a distance of 10,000 base pairs (kb) to ensure independence among the included SNPs. For consistency, we harmonized exposures and outcomes in terms of the effect allele and carried out subsequent analyses using the merged exposure-outcome dataset.

### Power analysis

The F statistic is a measure of instrument strength that is related to the proportion of variance in the phenotype explained by the genetic variants (R^2^), sample size (N), and the number of instruments (k) by the formula F = R^2^(N − k − 1)/k(1 − R^2^) [13]. And R^2^ can be calculated by the formula R^2^ = 2*Effect^2^*EAF*(1-EAF)/[2*Effect^2^*EAF*(1-EAF) + SE^2^*2*N*EAF*(1-EAF)] [14]. An F statistic of ≥ 10 indicates a relatively low risk of weak instrument bias in MR analysis [14].

### Statistical analysis

We utilized five well-established MR methods, comprising inverse-variance weighted (IVW), MR-Egger regression, weighted median, weighted mode, and simple mode, to analyze data involving multiple IVs. The primary emphasis was placed on the IVW method for our main results, with the other methods providing supplementary insights. To gauge the heterogeneity among IVs, we employed Cochrane’s Q-statistic, considering *p* < 0.05 as indicative of significant heterogeneity. In the presence of notable pleiotropy, we conducted the MR-Egger intercept test to assess directional pleiotropy based on the intercept [15]. To assess result stability, we conducted a leave-one-out sensitivity analysis, systematically excluding individual IVs one at a time [16]. All statistical analyses were performed using R packages, including two-sample MR and MR-PRESSO.

## Results

**Fig. 1:**
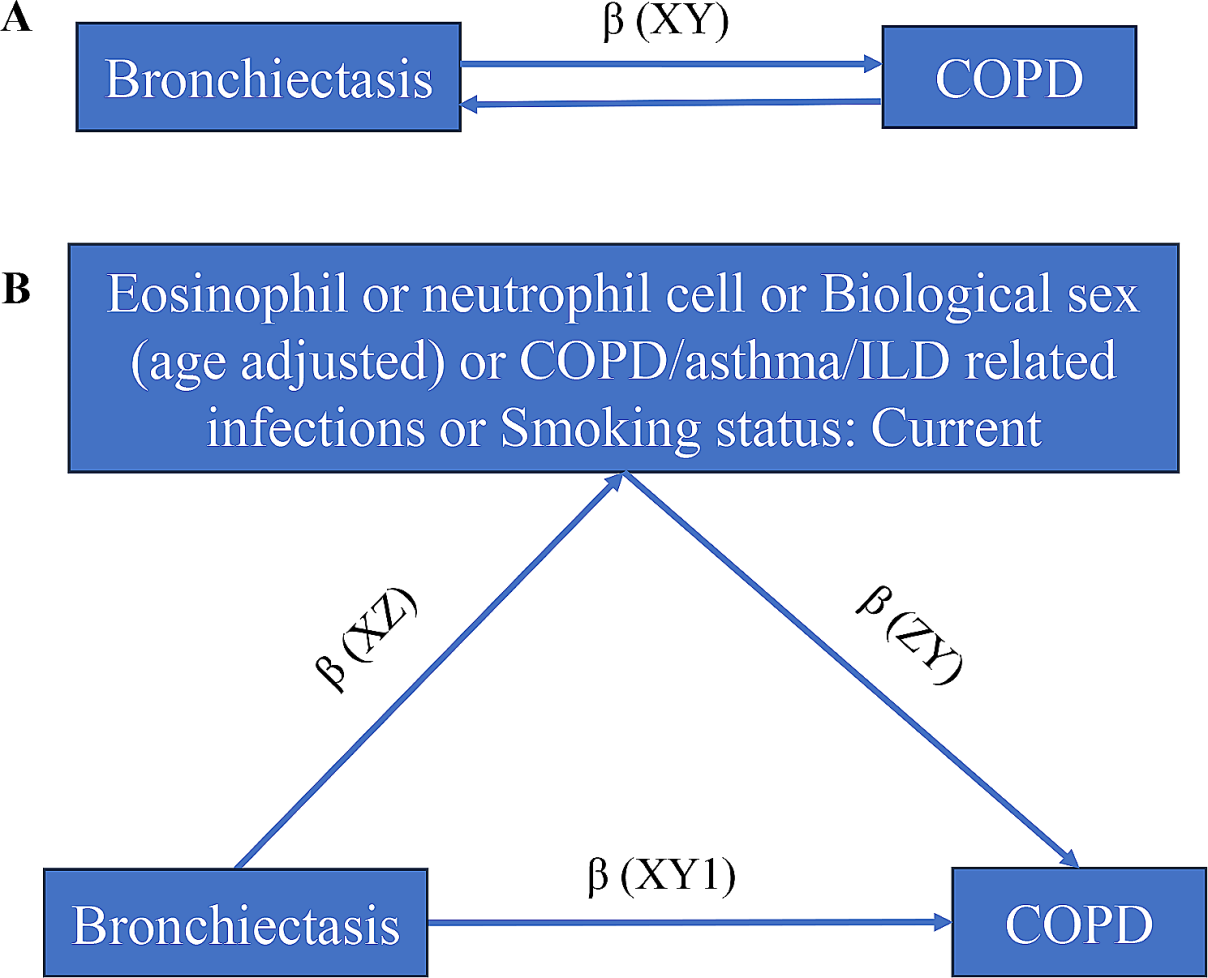
Illustration of the total effect and direct effect of bronchiectasis on COPD. (**A**) The total effect of bronchiectasis on COPD is equal to β(XY). (**B**) The direct effect of bronchiectasis on COPD is the effect bronchiectasis has on COPD not via any other exposure variables, which is equal to β (XY1). The total effect bronchiectasis has on COPD is the effect of bronchiectasis on COPD directly plus the effect of bronchiectasis on COPD via eosinophil or neutrophil cell or biological sex (age adjusted) or COPD/asthma/ILD related infections or smoking status, which is equal to β(XY1) + β (XZ)*β(ZY). COPD, Chronic Obstructive Pulmonary Disease. ILD, Interstitial Lung Disease

A graphical summary of analyses is given in Fig. 1. Initially, we conducted univariable two-sample MR to estimate the total effect of bronchiectasis on COPD (β (XY) in Fig. 1A), and the total effect of COPD on bronchiectasis, as well as the effect of bronchiectasis on each mediator (β (XZ) in Fig. 1B). Subsequently, we employed multivariable MR (MVMR) to estimate the effect of each mediator on COPD (β (ZY) in Fig. 1B) while accounting for the influence of bronchiectasis. The total effect of an exposure on an outcome can be dissected into indirect effects (i.e., β (XZ)*β (ZY) in Fig. 1B, representing the influence of bronchiectasis on COPD mediated through blood cells, smoking status, COPD/asthma/ILD related infections) and direct effects (i.e., β (XY1) in Fig. 1B, signifying the impact of bronchiectasis on COPD after adjusting for each mediator) [17].

In our MR analysis, we utilized SNPs as IVs classified as “strong” instruments, each with an F statistic exceeding 10 [18]. The F statistic takes into account both the strength and precision of a SNP’s association with COPD. The individual F statistics for these instruments ranged from 21.00 to 1535.09. For further details regarding these genetic variants, please refer to Supplementary Tables 2–11.

### Effects of COPD on bronchiectasis

**Fig. 2:**
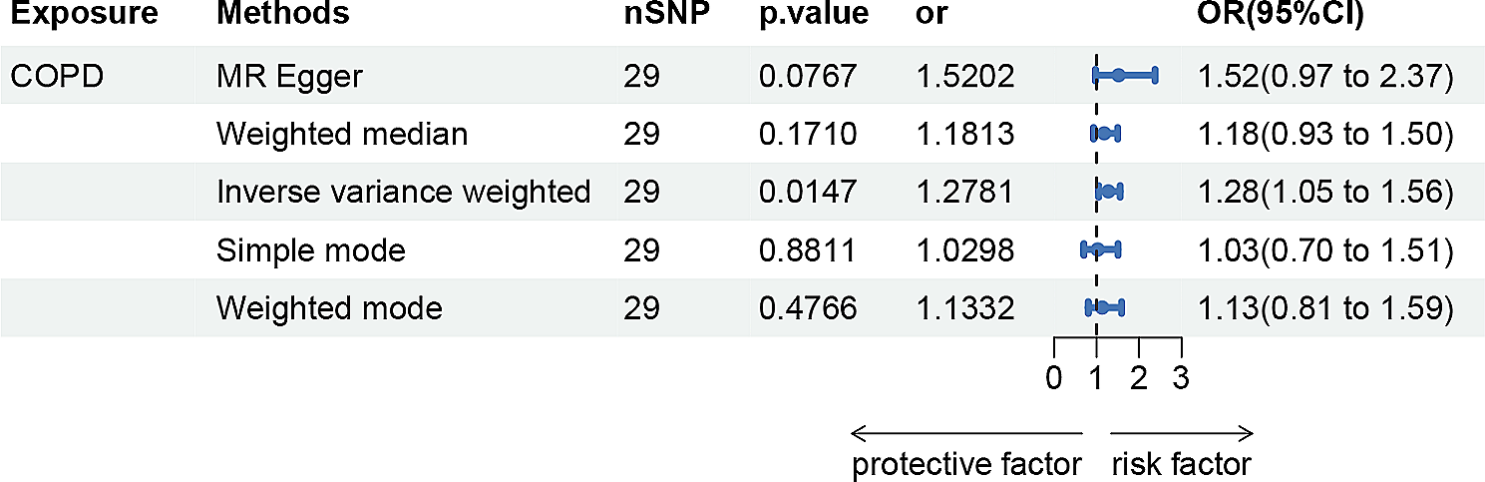
MR estimates of COPD on bronchiectasis. COPD, Chronic Obstructive Pulmonary Disease

As shown in Fig. 2, genetically predicted COPD causally led to a 1.28-fold increase in bronchiectasis risk [95% confidence interval (CI)] = 1.05–1.56, *p* = 0.0147 for the IVW estimator. In sensitivity analyses, there was statistical evidence of heterogeneity evaluated by Cochran’s Q test statistics with a *p*-value of 0.046 and 0.047 for MR Egger and IVW. The pleiotropic effect was detected by the MR-Egger intercept, and there were no signs of pleiotropy for bronchiectasis (MR-Egger: intercept term = − 0.0292; *p* = 0.402) (Supplementary Table 12).

### Effects of bronchiectasis on COPD, blood cell traits, biological sex (age adjusted), smoking status, COPD/asthma/ILD related infections

**Fig. 3:**
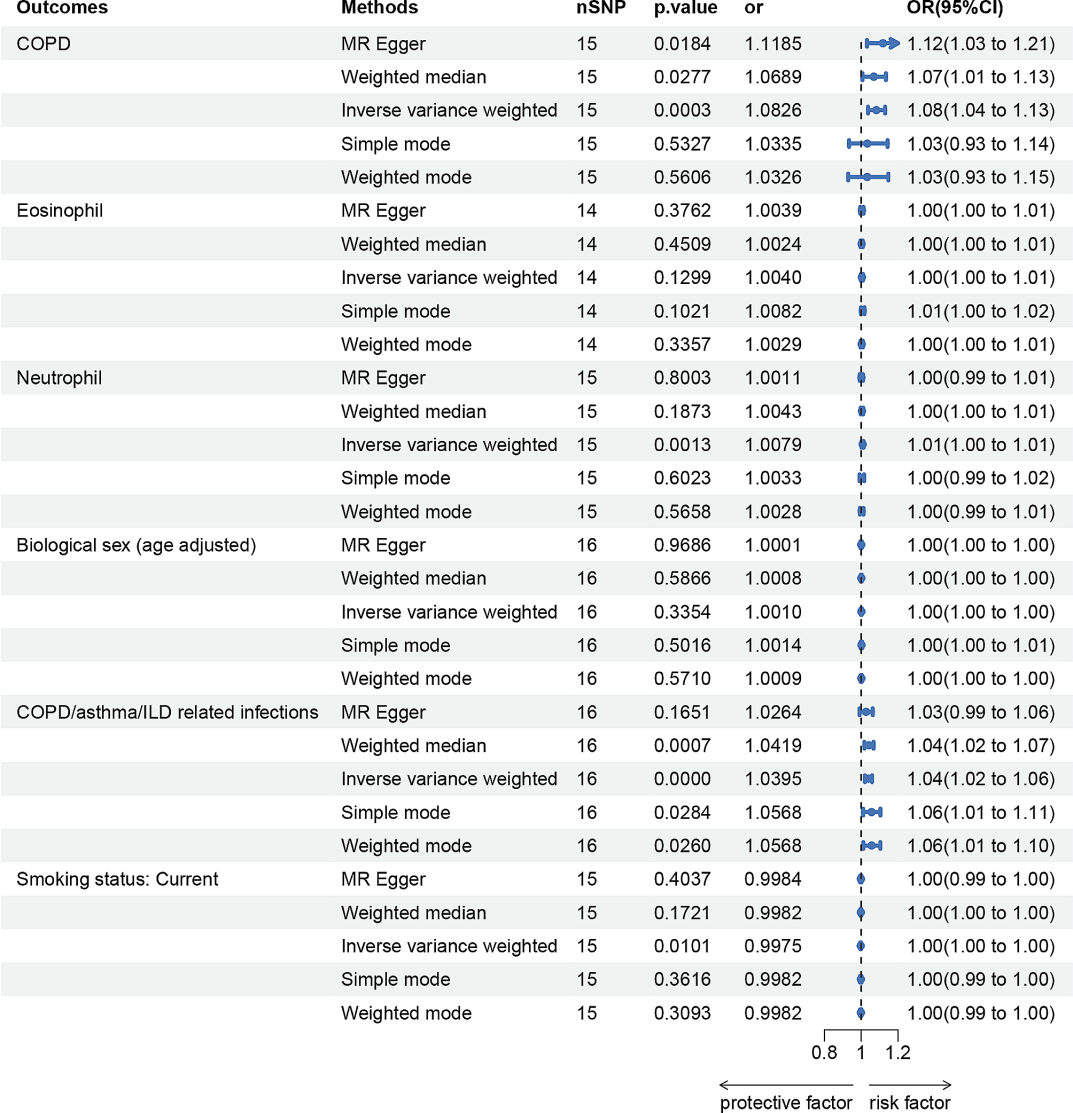
MR estimates of bronchiectasis on COPD, eosinophil cell, neutrophil cell, biological sex (age adjusted), COPD/asthma/ILD related infections and smoking status. COPD, Chronic Obstructive Pulmonary Disease. ILD, Interstitial Lung Disease

As shown in Fig. 3, genetically predicted bronchiectasis causally led to a 1.08-fold increase in COPD risk (95% CI) = 1.04–1.13, *p* = 0.0003), bronchiectasis causally led to a 1.01-fold increase in a higher level of neutrophil counts (95%CI = 1.00–1.01, *p* = 0.0013), bronchiectasis causally led to a 1.04-fold increase in COPD/asthma/ILD related infections (95%CI = 1.02–1.06, *p* = 0.0000) and lower risk of smoking for the IVW estimator. There was consistent support across MR Egger and Weighted median for a causal effect of bronchiectasis on higher COPD risk. And there was consistent support across weighted median, weighted mode, and simple mode for a causal effect of bronchiectasis on higher COPD/asthma/ILD related infections risk. However, there is no causal relationship among eosinophils, biological sex and COPD.

In sensitivity analyses, there was some statistical evidence of heterogeneity evaluated by Cochran’s Q test statistics, with a *p*-value < 0.05 for MR Egger in analyzing bronchiectasis on eosinophils. The pleiotropic effect was detected by the MR-Egger intercept, and there were no signs of pleiotropy for all outcomes. Detailed information about the sensitivity analyses of MR in this study is provided in Supplementary Table 12.

### Effects of potential mediator on COPD

**Fig. 4:**
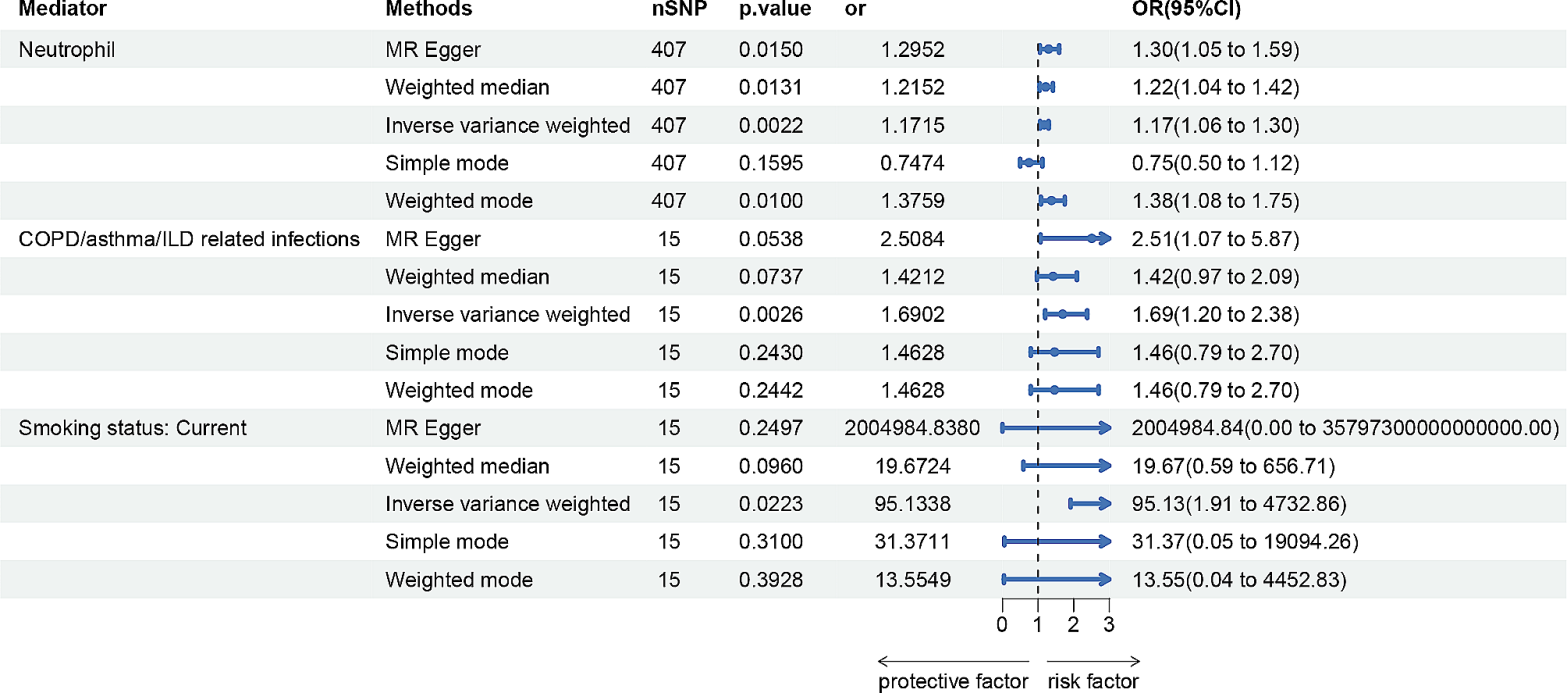
MR estimates of neutrophil cell, COPD/asthma/ILD related infections and smoking status on COPD. COPD, Chronic Obstructive Pulmonary Disease. ILD, Interstitial Lung Disease

The higher neutrophil counts causally led to a 1.17-fold increase in COPD risk (95%CI = 1.06–1.30, *p* = 0.0022), COPD/asthma/ILD related infections causally led to a 1.69-fold increase in COPD risk (95%CI = 1.20–2.38, *p* = 0.0026), smoking causally led to a 95.13-fold increase in COPD risk (95%CI = 1.91–4732.86, *p* = 0.0223) for the IVW estimator (Fig. 4). There was broadly consistent support across most MR methods for a positive effect of neutrophil counts on COPD risk. In sensitivity analyses, there was some statistical evidence of heterogeneity evaluated by Cochran’s Q test statistics in analyzing neutrophil counts and COPD/asthma/ILD related infections on COPD, and there were two significant outliers detected in analyzing smoking status on COPD. The pleiotropic effect was detected by the MR-Egger intercept, and there were no signs of pleiotropy among neutrophil counts, COPD/asthma/ILD related infections, smoking status and COPD. Detailed information about the sensitivity analyses of MR in this study is provided in Supplementary Table 12.

### Mediating effects of neutrophil cells, COPD/asthma/ILD related infections, smoking status on bronchiectasis–COPD effects

**Fig. 5:**
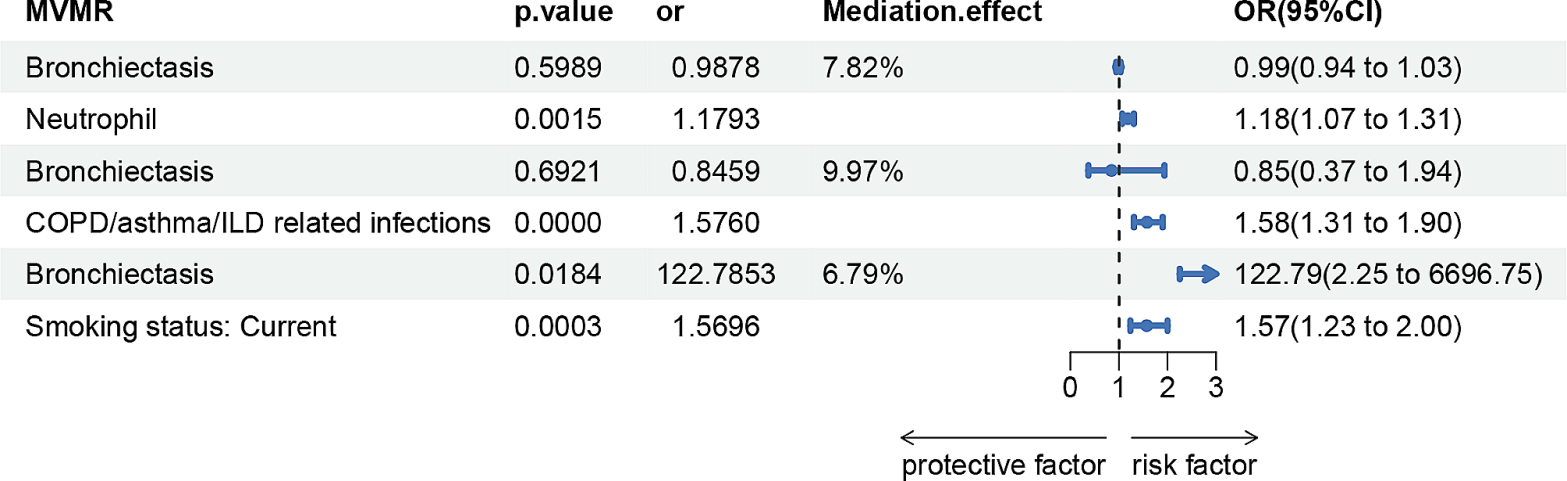
The mediation effect of bronchiectasis on COPD via neutrophil cells, COPD/asthma/ILD related infections and smoking status by Multivariate MR analysis. COPD, Chronic Obstructive Pulmonary Disease. ILD, Interstitial Lung Disease

We explored the potential mediator that had causal support from MR for both an effect of bronchiectasis on them (step one) and of the mediator on COPD (step two): neutrophil cells, COPD/asthma/ILD related infections, smoking status. The bronchiectasis–COPD effect decreased from 1.08 (95% CI 1.04, 1.13) to 0.99 (95% CI 0.94, 1.03) with adjustment for the estimated effects of neutrophil, decreased from 1.08 (95% CI 1.04, 1.13) to 0.85 (95% CI 0.37, 1.94) with adjustment for the estimated effects of COPD/asthma/ILD related infections, and increased from 1.08 (95% CI 1.04, 1.13) to 122.79 (95% CI 2.25, 6696.75) with adjustment for the estimated effects of smoking status (Fig. 5). The bronchiectasis–COPD effect was 0.99, 0.85 and 122.79 with genetic adjustment for neutrophils, COPD/asthma/ILD related infections and smoking. Our results suggested that smoking was an important mediator, contributing to the mediation of bronchiectasis on COPD.

## Discussion

We undertook a bidirectional two-sample MR study to investigate the causal relationship between bronchiectasis and COPD, and the results suggested a causal relationship between genetically predicted bronchiectasis and increased risk of COPD and COPD was also associated with a higher risk of bronchiectasis. Our findings augment the growing body of evidence indicating that individuals with bronchiectasis face an elevated risk of developing COPD, offering a deeper comprehension of the role of neutrophil cells, respiratory infections and smoking status in linking these intricate respiratory conditions. The established causal link between bronchiectasis and COPD aligns with prior epidemiological investigations that have suggested an association between these two diseases [5, 6]. Importantly, the data from Huang et al. [8] revealed that the fundamental pathophysiology of the “COPD-bronchiectasis association” bears a closer resemblance to that of bronchiectasis, which also supported bronchiectasis as a cause of COPD. Meanwhile, Bronchiectasis has been categorized as a comorbidity associated with COPD, exerting a notable influence on the inherent progression and prognosis of COPD. Roughly 30% of individuals diagnosed with COPD also experience the impact of bronchiectasis [8]. A significant study conducted in the United Kingdom revealed that 30% of individuals within a primary-care population diagnosed with COPD exhibited airway wall abnormalities that could be potentially categorized as indicative of bronchiectasis [5]. Our MR analysis contributes robust evidence by utilizing genetic variants as IVs, mitigating potential biases inherent in observational studies. This approach reaffirms the notion that bronchiectasis and COPD are mutually causal. Especially, bronchiectasis acts as a risk factor for COPD, underscoring the importance of recognizing bronchiectasis as a potential precursor in the development of COPD. The frequency of airway wall thickening and dilation meeting the criteria for bronchiectasis rises proportionally with the escalating spirometric severity of COPD.

Although bronchiectasis and COPD typically manifest distinct clinical characteristics, a subset of patients exhibits features of both conditions, suggesting the potential existence of a shared inflammatory pattern. This overlap in characteristics is often referred to as bronchiectasis-COPD overlap [4, 7]. The mechanistic foundations of the bronchiectasis-COPD relationship are intricate, with airway inflammation emerging as a central mediator. Our study lends support to the hypothesis that the increased vulnerability of bronchiectasis patients to the development of COPD can be attributed to the presence of persistent airway inflammation. Pseudomonas infections are often found in bronchiectasis, which is associated with an increased risk of airway inflammation. Neutrophilic inflammation plays a pivotal role in the pathophysiology of several chronic lung conditions [19]. The emergence of neutrophil extracellular traps (NETs) has been recognized as a critical disease mechanism in neutrophilic lung disorders, such as COPD and bronchiectasis [20]. This inflammation, mediated by neutrophils, acts as a bridge connecting the two diseases.

The recognition of neutrophil-mediated airway inflammation as a mechanistic bridge connecting bronchiectasis and COPD highlights potential therapeutic implications. This revelation could influence treatment decisions, underscoring the importance of selecting therapies that target neutrophilic inflammation, irrespective of the clinical diagnosis. Strategies aimed at addressing airway inflammation hold promise not only in alleviating bronchiectasis symptoms but also in potentially halting or delaying the progression of COPD. Interventions focused on modulating neutrophil activity may disrupt the sequence of events leading to COPD development in susceptible individuals.

In our MR study, we observed a relatively small odds ratio (OR) with statistical significance in analyzing bronchiectasis on neutrophil cell counts and smoking status. While the effect size is small, the findings remain statistically robust. The possible reason is that the included patients with bronchiectasis are in a stable stage and the airway inflammation is not serious. Secondly, patients with bronchiectasis who usually smoke are more likely to choose to quit smoking and take anti-inflammatory medications for health reasons, which seemingly minor effect size. Although the OR is small, its statistical significance contributes valuable insights to the understanding of the clinical implications.

However, it’s essential to acknowledge the limitations of our study. Our datasets primarily originated from the European population, which somewhat limits the generalizability of our findings. And all the data came from online Open GWAS database, which do not provide additional clinical information about exposures and outcomes included. In particular, the lack of a clear definition of COPD and bronchiectasis affected the interpretation of the results. The utilization of summary statistics, instead of raw data, in the research rendered it challenging to address the issue of potential confounding due to infective exacerbations in COPD and bronchiectasis patients at the time of blood draw. Furthermore, while we have unveiled a mechanistic pathway involving airway inflammation, smoking and respiratory infection, it’s important to recognize that other factors may also contribute to the connection between bronchiectasis and COPD, such as age, occupation and environment. Further research is warranted to comprehensively explore the intricate interplay among genetic predisposition, immune response, and environmental factors within the context of bronchiectasis and COPD.

Our MR study provides robust evidence for the causal relationship between bronchiectasis and COPD, shedding light on a potential pathway. This pathway suggests that COPD patients were associated with a higher risk of bronchiectasis, and bronchiectasis patients, characterized by airway inflammation driven by neutrophils, may have an increased predisposition to developing COPD. These findings underscore the importance of proactive monitoring and management of individuals with COPD and bronchiectasis to reduce the risk of subsequent bronchiectasis and COPD development. Especially, patients with COPD are more likely to develop bronchiectasis than patients with bronchiectasis to suffer from COPD. These results could suggest that anti-neutrophil agents, anti-infective drugs and smoking cessation may be of value in people with features of bronchiectasis and COPD.

### Electronic supplementary material

Below is the link to the electronic supplementary material.

Supplementary Material 1

## Data Availability

The data is available on the IEU OpenGWAS database (https://gwas.mrcieu.ac.uk/).
